# Bottom-up plasma-enhanced atomic layer deposition of SiO_2_ by utilizing growth inhibition using NH_3_ plasma pre-treatment for seamless gap-fill process

**DOI:** 10.1038/s41598-022-20201-y

**Published:** 2022-09-21

**Authors:** Yoenju Choi, Taehoon Kim, Hangyul Lee, Jusung Park, Juhwan Park, Dongho Ryu, Woojin Jeon

**Affiliations:** 1 Department of Advanced Materials Engineering for Information and Electronics, and Integrated Education Program for Frontier Science and Technology (BK21 Four), Kyung Hee University, Yongin, Gyeonggi 17104 Korea; 2 Semiconductor R&D Center, WONIK IPS Co., Ltd., Pyeongtaek, Gyeonggi 17709 Korea

**Keywords:** Electrical and electronic engineering, Techniques and instrumentation, Materials for devices, Techniques and instrumentation

## Abstract

The design-rule shrinkage in semiconductor devices is a challenge at every step of the integration process. In the gap-fill process for isolation, the seam and void formation cannot be suppressed by using a deposition process, which even has excellent step coverage. To achieve seamless gap fill in the high-aspect-ratio structure, which has a non-ideal etch profile such as a negative slope, the deposition process should be able to realize the “bottom-up growth” behavior. In this work, the bottom-up growth of a SiO_2_ plasma-enhanced atomic layer deposition (PE-ALD) process in a trench structure was investigated by using a growth inhibition process employing plasma treatment. N_2_ and NH_3_ plasma pre-treatments were employed to suppress the growth of the SiO_2_ PE-ALD process without any contamination, and the inhibition mechanism was investigated by performing surface chemistry analyses using X-ray photoelectron spectroscopy. Furthermore, the gap-fill characteristics of the SiO_2_ PE-ALD process were examined, depending on the process conditions of NH_3_ plasma pre-treatment, by performing cross-sectional field emission scanning electron microscopy measurements. Finally, a seamless gap-fill process in a high-aspect-ratio trench pattern was achieved by the bottom-up growth behavior of SiO_2_ PE-ALD using NH_3_ plasma pre-treatment.

## Introduction

Design rule shrinkage is a crucial requirement for increasing the integration density in semiconductors. To achieve a device with a reduced design rule, not only the patterning processes, such as lithography and etching, but also the isolation between actives, where the channel is located, is important to ensure robust device operation^1–6^. In this regard, isolation processes, such as shallow trench isolation, contact holes, channel hole oxides, and inter-metal dielectrics, have become important for obtaining cutting-edge semiconductor devices^2,7–10^. However, the difficulty of the isolation process increases with an increased aspect ratio of the structure and decreased space between actives (less than tens of nanometers) having a fixed depth (~ 1 μm). Therefore, a deposition process with excellent step coverage capability that can fill the space between actives without seam or void formation should be developed: this is one of the characteristics of the deposition process and is called “gap-fill characteristics.” In this regard, atomic layer deposition (ALD) has been employed as a gap-fill process using SiO_2_ thin films^2,7–15^. The ALD process exhibits self-limiting growth behavior induced by the chemisorption of a precursor at the surface of the substrate, resulting in a step coverage of over 95%. However, the excellent step coverage of the ALD process induces the formation of seams or voids^9,14^. In the case of a high-aspect-ratio pattern with an ideal etch profile and a positive slope, the ALD process can fill the structure. However, the real etch profile of a high-aspect-ratio pattern would have a negative slope, resulting in tapering or bowing shapes owing to process difficulty^7,16^. Where the sidewall of the trench or hole has a negative slope, the conformal SiO_2_ film deposition induces the formation of seams or voids. In other words, the excellent step coverage characteristics of the deposition process hinder the gap-fill characteristics.

Therefore, in the high-aspect-ratio pattern, higher growth rate at the bottom region of the trench than at the top region and surface would be favorable for filling the pattern without the formation of seams or voids. This is called “bottom-up growth”^2,17,18^. However, most of the deposition processes have a relatively higher growth rate in the top region of the trench (“top region”) because the concentration of the reactant is higher in this region^19^. Hence, the introduction of an inhibitor, which can suppress the growth of thin film deposition, was investigated to control the growth rate in this region. Among the various inhibition techniques, the plasma process is adequate for demonstrating the inhibition effect only in the top region^20,21^, because the plasma generally has a large concentration gradient in the high-aspect-ratio pattern^19,22^. However, plasma treatment can induce N or C contamination depending on the gas species, which can contribute to defects in the thin film.

Based on these considerations, the bottom-up growth of SiO_2_ thin film deposition by employing a surface modification process via plasma treatment for the gap-fill process was investigated in this work. Plasma treatment was conducted by employing gases having a simple structure, namely, N_2_ and NH_3_, to avoid contamination. The inhibitory effect of the plasma treatment on the growth of the SiO_2_ thin film was examined. Moreover, the mechanism involved in this inhibitory effect was evaluated. Eventually, the bottom-up growth behavior and gap-fill characteristics were investigated by employing plasma pre-treatment.

## Results and discussion

**Figure 1:**
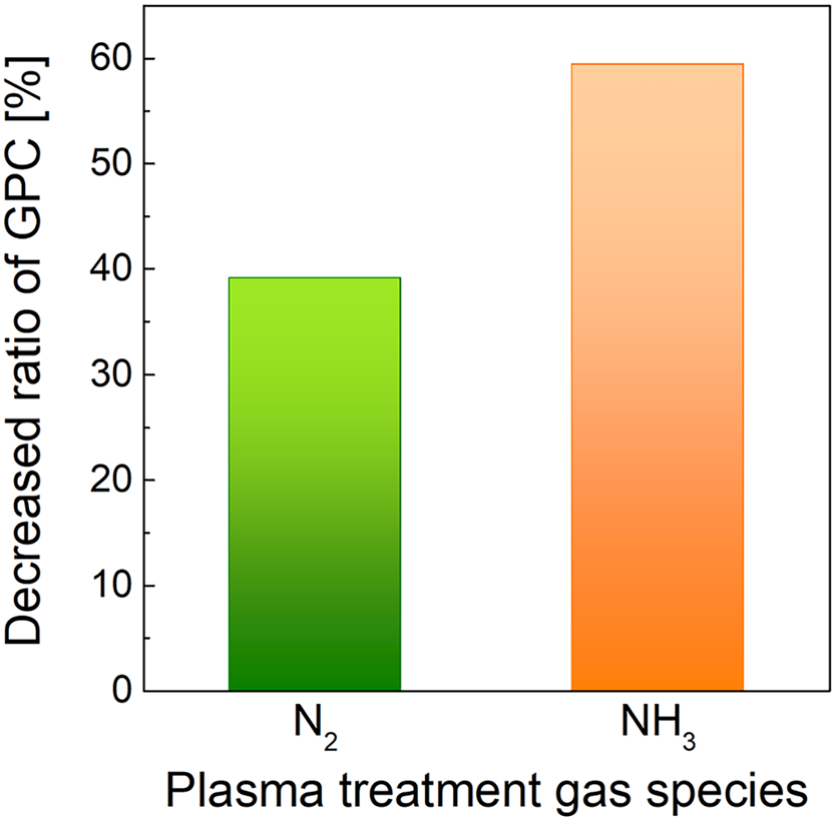
Decreased GPC ratio of SiO_2_ PE-ALD with N_2_^*^ and NH_3_^*^ compared with the no inhibitor case.

Figure 1 and Table 1 present the growth inhibition effect of plasma pre-treatment depending on the gas species, namely, N_2_ and NH_3_. Before the plasma-enhanced ALD (PE-ALD) SiO_2_ sequence was implemented, 1 s of plasma pre-treatment was performed for each gas species. The growth rate (growth per cycle, GPC) of PE-ALD SiO_2_ was 0.064 nm/cycle without any plasma pre-treatment, and it decreased to 0.039 and 0.026 for N_2_ plasma pre-treatment (N_2_^*^) and NH_3_ plasma pre-treatment (NH_3_^*^), respectively. The decreased GPC ratios compared with the case of no inhibitor were 39.1, and 59.4% for N_2_^*^ and NH_3_^*^, respectively. The difference in the inhibition effect was attributed to the difference in the reactivity between N_2_^*^ and NH_3_^*^. As NH_3_ consists of relatively weak chemical bonding, N–H, a relatively large number of radicals might be induced, resulting in higher growth inhibition in the PE-ALD SiO_2_.

**Table 1:** GPC of SiO_2_ PE-ALD with various inhibition conditions and decreased ratio compared with the no inhibitor case.

Gas species	GPC (nm/cycle)	Decreased ratio (%)
No inhibitor	0.064	
N_2_	0.039	39.1
NH_3_	0.026	59.4

**Figure 2:**
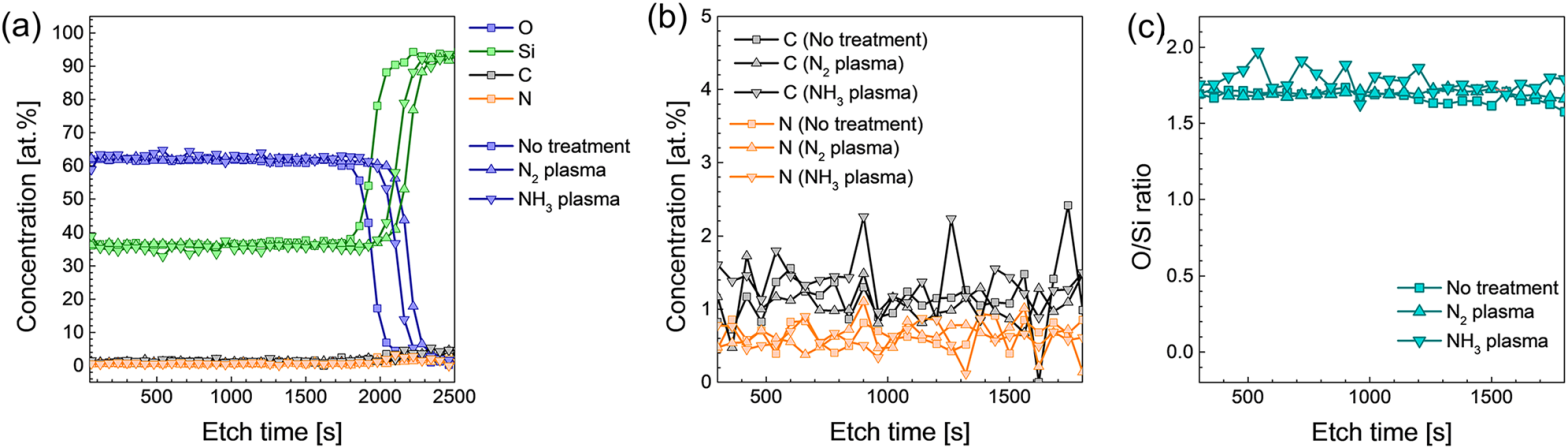
(**a**) Depth profiles of O, Si, C, and N; (**b**) depth profiles of C and N; (**c**) oxygen stoichiometry (O/Si ratio) of the thin films.

The chemical status and impurity incorporation during plasma pre-treatment were investigated using X-ray photoelectron spectroscopy (XPS). Figure 2a shows the depth profiles of deposited SiO_2_ thin films with no treatment, N_2_^*^, and NH_3_^*^. In all cases, the deposited SiO_2_ thin films had identical depth profiles for all elements, regardless of pre-treatment. To confirm the impurity incorporation by employing plasma pre-treatment, the depth profiles of C and N are depicted in Fig. 2b, which is a magnification of those in Fig. 2a. As shown in Fig. 2b, the concentrations of both C and N are less than 2 at.%, which is the detection limit of the XPS measurement. In addition, no increment in C or N concentration is observed by employing plasma pre-treatment. Because of the almost identical depth profiles and negligible C and N concentrations, plasma pre-treatment can inhibit the growth of SiO_2_ PE-ALD without inducing any contamination or residue. Moreover, the stoichiometry of the deposited SiO_2_ thin films was calculated (Fig. 2c). In the case of SiO_2_ without pre-treatment, the O/Si ratio was 1.68 with homogeneity in the depth direction (standard deviation of 0.033). A relatively low O/Si ratio was attributed to the different sputtering yields between Si and O (lighter atom tends to easily sputtered out) during the etching for the depth profiling. The SiO_2_ thin film with N_2_^*^ had an O/Si ratio of 1.70 with a high uniformity (standard deviation of 0.017). In contrast, the O/Si ratio in the SiO_2_ thin film with NH_3_^*^ was relatively high at 1.78, with a slightly large fluctuation in the depth direction and a standard deviation of 0.075.

**Figure 3:**
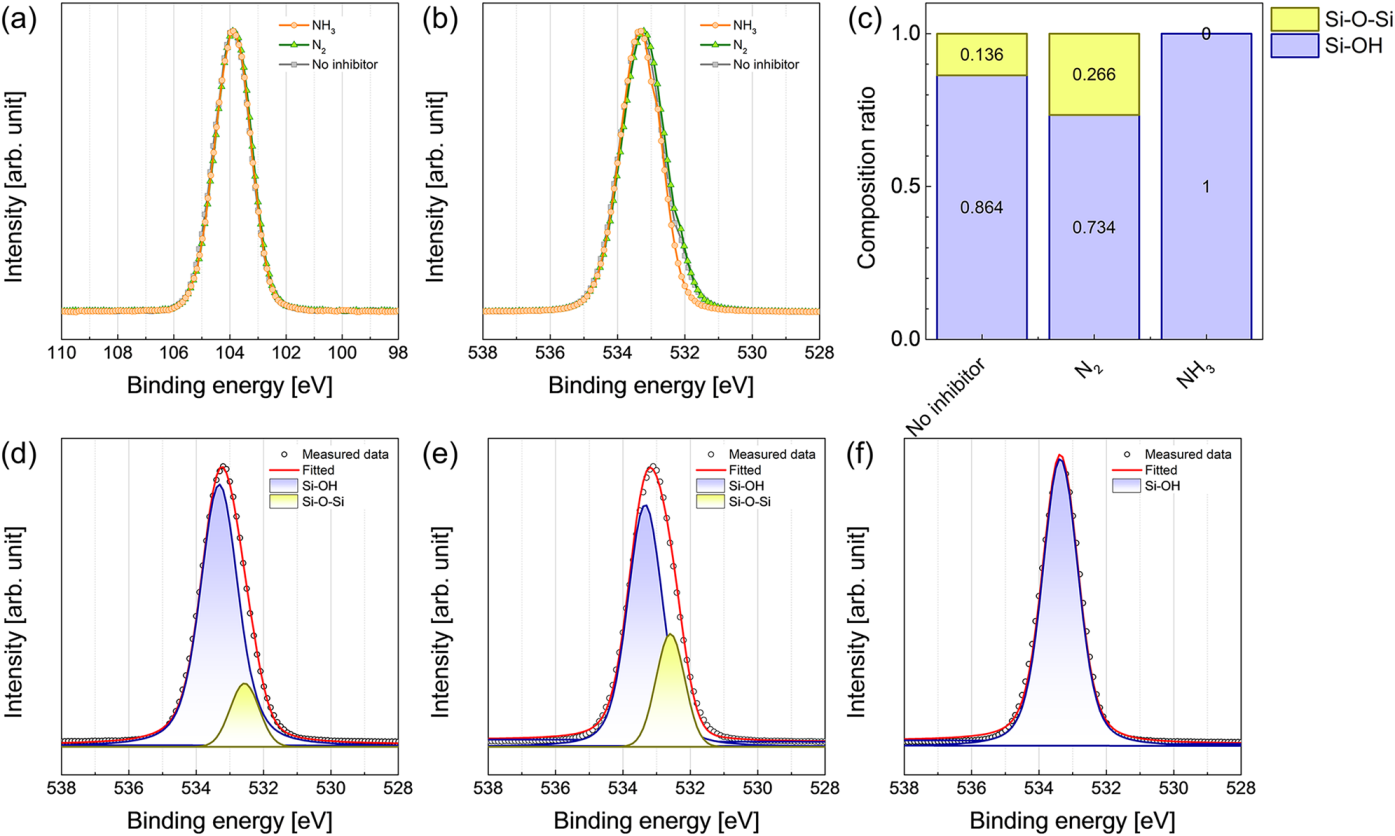
XPS profiles of (**a**) Si2*p*, and (**b**) O1*s* of SiO_2_ thin films. (**c**) Composition ratio of Si–O–Si and Si–OH bonding in O1*s* XPS spectra. Deconvoluted XPS O1*s* peaks of SiO_2_ thin films with (**d**) no inhibitor, (**e**) N_2_^*^, and (**f**) NH_3_^*^.

The chemical states of the deposited films were also investigated. Figure 3a shows the Si2*p* XPS spectra of the SiO_2_ thin films. All the spectra have identical profiles and only consist of Si–O bonding, corresponding to a binding energy of 103.8 eV^23^. However, in the O1*s* XPS spectra, as shown in Fig. 3b, a difference is observed depending on the plasma pre-treatment. In the case of no inhibitor and N_2_^*^, a shoulder at approximately 532 eV is observed, which is evidence that the peak at the lower binding energy contributed to the spectra. From the O1*s* spectra, the composition of two bondings on the surface was evaluated (Fig. 3c) by the deconvolution to peaks at 532.6 and 533.5 eV corresponding to the Si–O–Si and Si–OH bonding, respectively (Fig. 3d–f)^23^. These two bonds are strongly related to the SiO_2_ PE-ALD process. The Si–O–Si bond corresponds to the covalent bond of SiO_2_. The Si–OH bonding is the surface termination of the SiO_2_ thin film. In the ALD process, the precursor chemisorbs on the surface through a ligand exchange reaction with the functional group on the surface, which is called “anchoring site,” and only the chemisorbed precursor participates in the film formation reaction with the reactant, resulting in the self-limiting growth characteristics. The surface functional groups and their chemistry and density strongly influence the growth behavior in the ALD process. In the SiO_2_ PE-ALD process, the Si–OH bonding, generally called the hydroxyl group, contributes to the ligand exchange reaction with diisopropylamino silane (DIPAS) (Si precursor)^15,24^. The Si–OH bonding on the surface is changed to Si_(from the surface)_–O–Si_(from the DIPAS)_–H_3_ during the precursor feeding step^24^. After the reactant feeding step and the reactant purge step, the Si–OH bonding on the surface is recovered and participates in the chemisorption of DIPAS. Therefore, after the SiO_2_ PE-ALD process is conducted, the surface consists of Si–OH bonds. In the case of SiO_2_ deposition with NH_3_^*^, the surface consists of only Si–OH bonding, implying that all the chemisorbed precursors fully react with the reactant and change into Si–OH bonding. Moreover, the passivated Si–OH bonding during the NH_3_^*^ step recovers to Si–OH bonding during the SiO_2_ PE-ALD process, resulting in growth inhibition without N contamination. In contrast to the case of NH_3_^*^, the no inhibitor and N_2_^*^ cases exhibit Si–O–Si bonding ratios of 0.136 and 0.266, respectively. The Si–O–Si bonding on the surface might originate from the remaining Si_(from the surface)_–O–Si_(from the DIPAS)_–H_3_ or the covalent bonding of SiO_2_. Both cases indicate that the recovery of the Si–OH bonding during the reactant feeding and purge steps was suppressed or insufficient.

**Figure 4:**
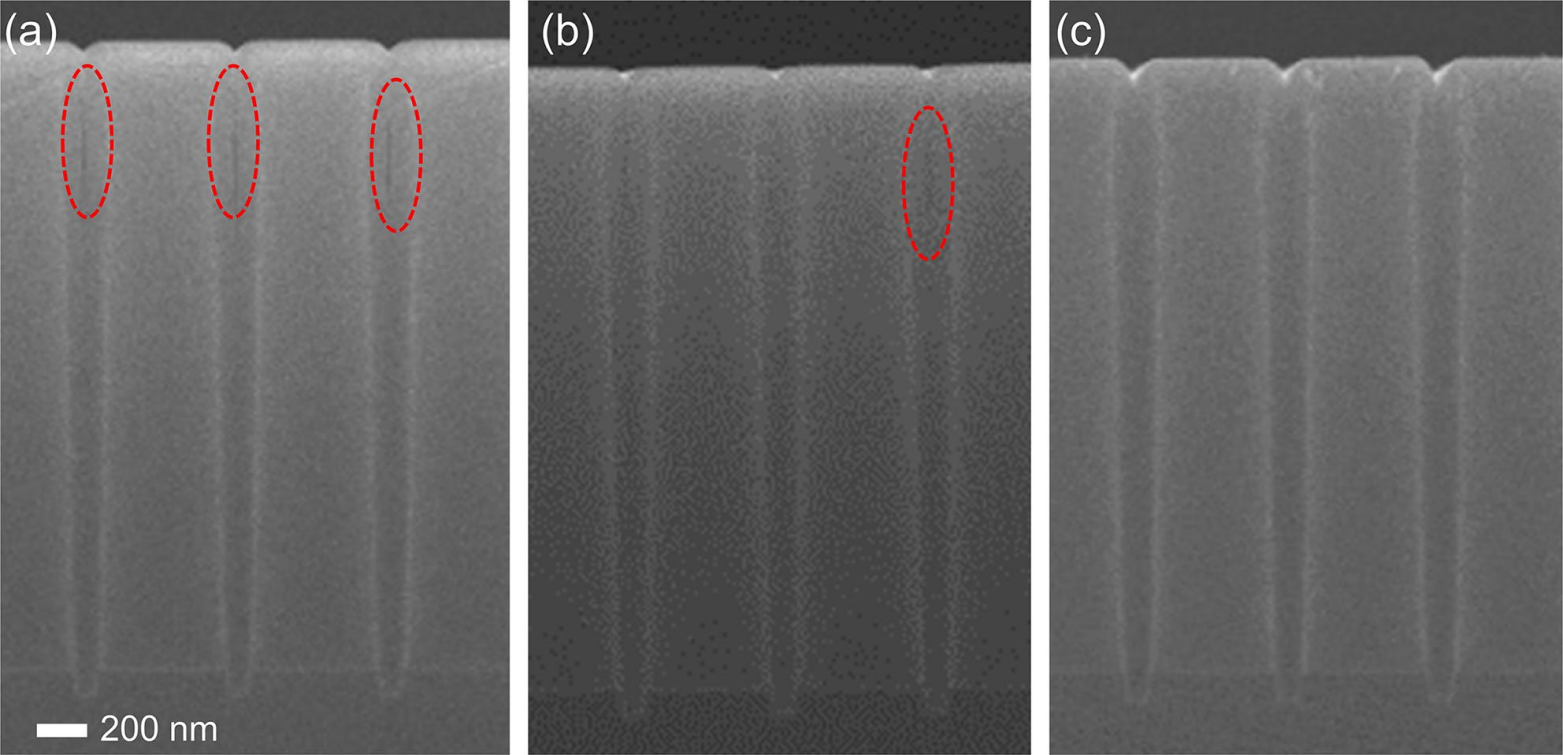
Cross-sectional field-emission scanning electron microscopy (FE-SEM) images of the trench pattern (aspect ratio of 25:1 with an opening size and a depth of 200 nm and 2.5 μm, respectively) after conducting SiO_2_ PE-ALD with (**a**) no inhibitor, (**b**) N_2_^*^, and (**c**) NH_3_^*^.

The gap-fill characteristics depending on the plasma pre-treatment were confirmed (Fig. 4). In the case of no inhibitor, voids in the trench are clearly observed, as shown in Fig. 4a. Although N_2_^*^ inhibits the growth of SiO_2_, the void formed by the imperfection gap fill remains (Fig. 4b). Only in the case of NH_3_^*^, a perfect gap-fill characteristic is obtained (Fig. 4c). Consequently, the inhibition of the growth of SiO_2_ can enhance the gap-fill characteristics.

**Figure 5:**
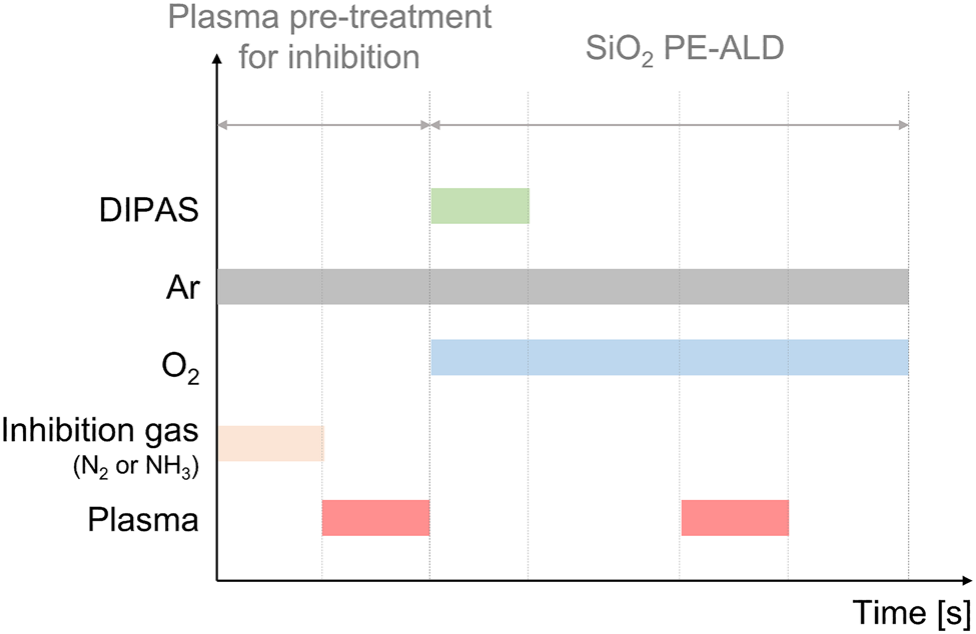
Schematic diagram of the process sequence constitution.

**Figure 6:**
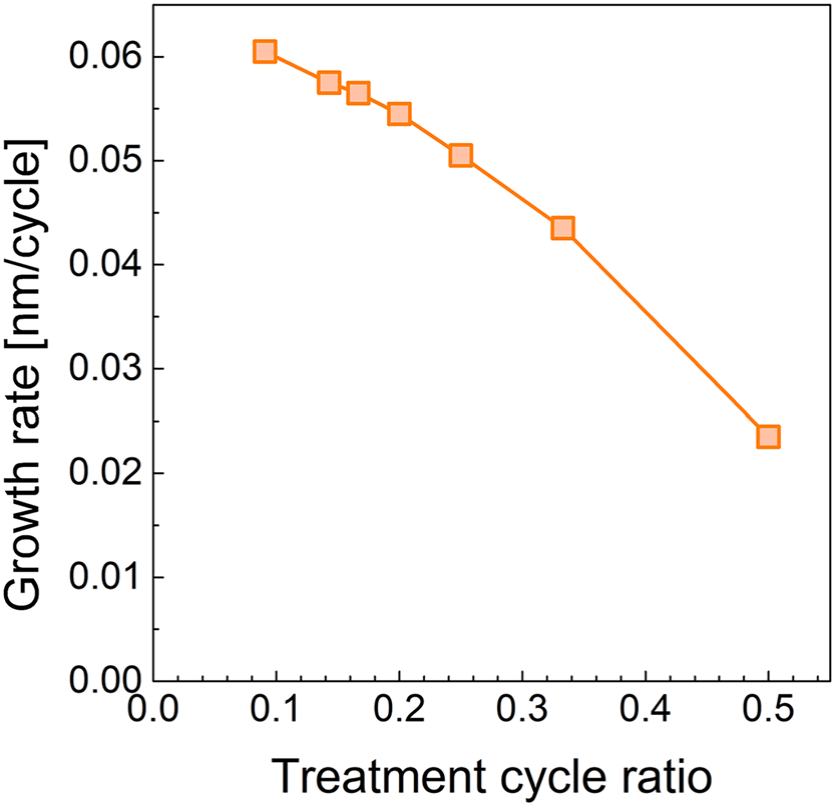
GPC change with respect to the treatment cycle ratio of the plasma pre-treatment to the total PE-ALD sequence as shown in Fig. 5.

**Figure 7:**
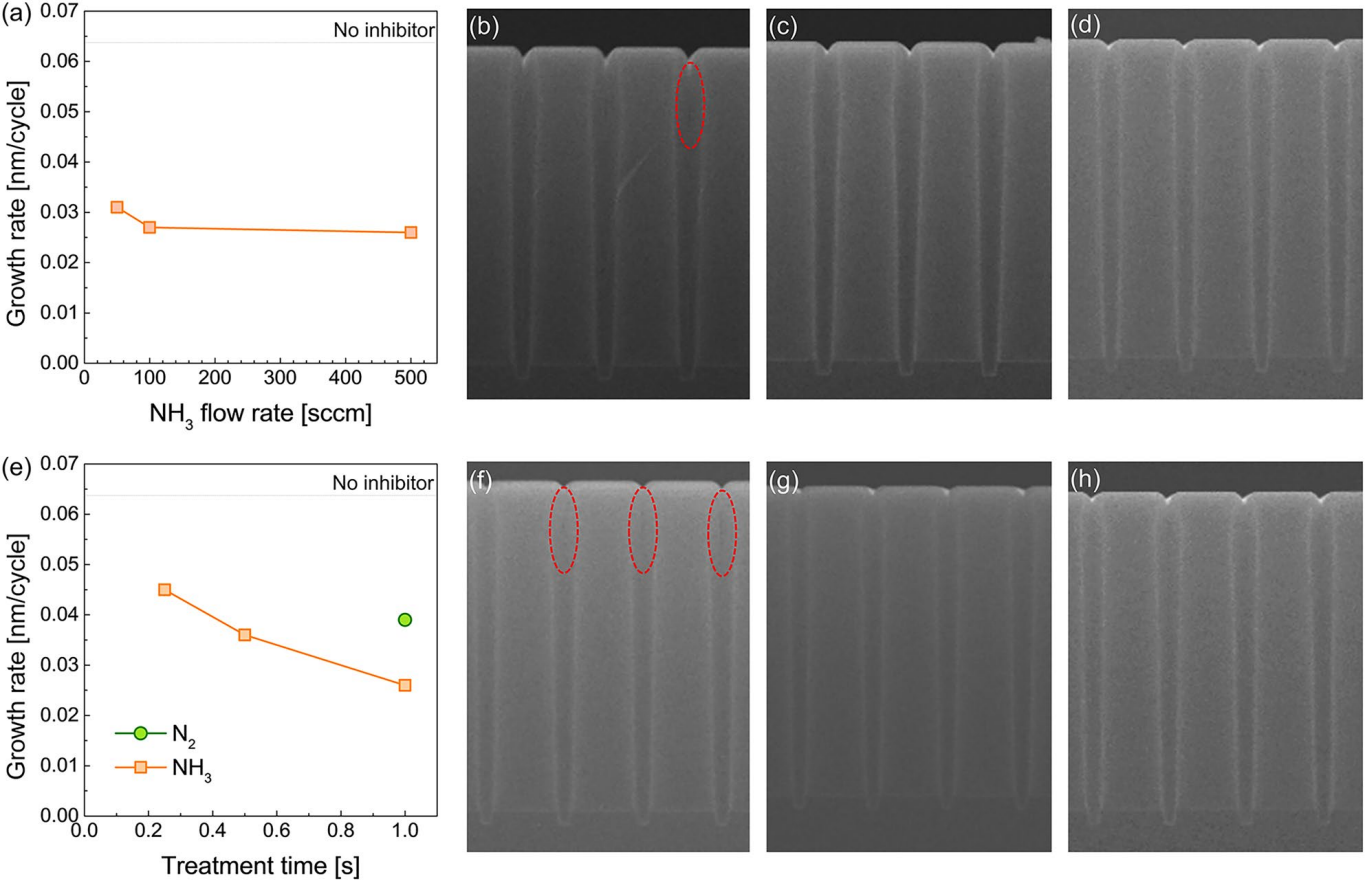
(**a**) GPC of SiO_2_ PE-ALD using NH_3_^*^ with various NH_3_ flow rates. Cross-sectional FE-SEM images of the trench pattern after deposition of SiO_2_ thin film using NH_3_^*^ with NH_3_ flow rate of (**b**) 50, (**c**) 100, and (**d**) 500 sccm. (**e**) GPC of SiO_2_ PE-ALD using NH_3_^*^ with various treatment times. N_2_^*^ with treatment time of 1 s is represented by the green dot for comparison. Cross-sectional FE-SEM images of the trench pattern after deposition of SiO_2_ thin film using NH_3_^*^ with treatment time of (**f**) 0.25, (**g**) 0.5, and (**h**) 1.0 s.

Further investigation of the growth inhibition effect by NH_3_^*^ was performed. First, growth inhibition depending on the ratio of NH_3_^*^ was evaluated. A treatment ratio of 0.1 indicates that the super-cycle consists of one cycle of NH_3_^*^ and nine cycles of SiO_2_ PE-ALD (refer to Fig. 5). In the same manner, a treatment ratio of 0.5 means a super-cycle consists of one cycle of NH_3_^*^ and one cycle of SiO_2_ PE-ALD. As shown in Fig. 6, the growth rate gradually decreases, displaying an almost linear relationship with an increasing treatment ratio, indicating that growth inhibition by employing NH_3_^*^ originated from the chemical inactivation of the Si–OH functional groups. The decreased Si–OH functional group density on the surface induces a decrease in the amount of chemisorbed Si precursor, resulting in a decrease in the growth rate. Moreover, the change in the growth rate depending on the NH_3_ flow rate during NH_3_^*^ was examined (Fig. 7a). The NH_3_ flow rate is related to the concentration of radicals formed in NH_3_^*^. When the NH_3_ flow rate was 50 standard cubic centimeter (sccm), the inhibition effect of NH_3_^*^ was slightly reduced from 0.026 nm/cycle to a growth rate of 0.031 nm/cycle. The decreased inhibition effect at an NH_3_ flow rate of 50 sccm induced a deteriorated gap-fill characteristic, as shown by the void formation in Fig. 7b. As the NH_3_ flow rate increasing to 100 sccm, the GPC was decreased to 0.026 nm/cycle, which is saturated value, and the seam-free gap-fill growth was observed (Fig. 7c,d). Furthermore, the time for NH_3_^*^ was varied at 500 sccm and the treatment ratio was 0.5. As shown in Fig. 7e, the growth rate increases from 0.026 nm/cycle to 0.36 and 0.45 nm/cycle as the treatment time decreases from 1.0 s to 0.5 and 0.25 s, respectively. The growth rate of 0.45 nm/cycle at a treatment time of 0.25 s is comparable to that of 0.039 nm/cycle for N_2_^*^. This relatively high growth rate results in defective gap-fill characteristics (Fig. 7f). In the case of the treatment time of 0.5 and 1.0 s, the seam was not observed (Fig. 7g,h). In this regard, the difference in the stoichiometry (Fig. 2c) and surface termination can be ascribed to the change in the amount of the chemisorbed precursor. Even though the reactant was overdosed during the ALD process, the stoichiometry of the deposited thin film was strongly influenced by the density of the chemisorbed precursor on the substrate^25^. The higher O/Si ratio in the SiO_2_ thin film prepared using NH_3_^*^ implies that the density of chemisorbed DIPAS decreased. Moreover, the recovery of surface termination to Si–OH (Fig. 3f) was facilitated by a relatively higher oxygen source ratio than the chemisorbed precursor.

**Figure 8:**
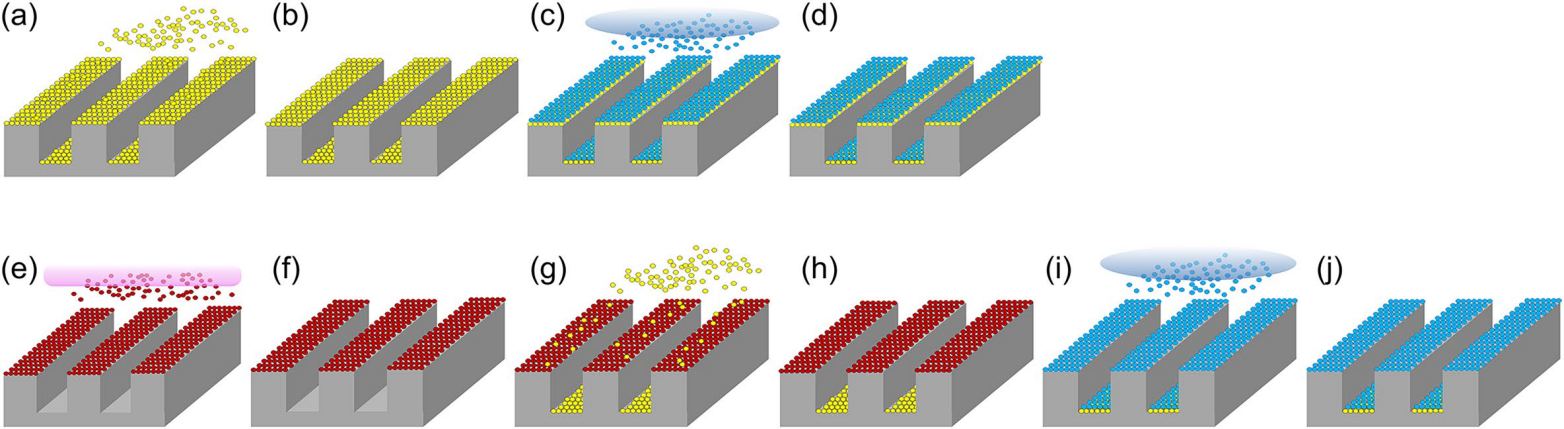
Schematic diagram of the SiO_2_ PE-ALD sequence consisting of (**a**) precursor feeding, (**b**) Ar purge, (**c**) O_2_ plasma, and (**d**) Ar purge. Schematic diagram of SiO_2_ PE-ALD using NH_3_^*^ sequence consisting of (**e**) NH_3_ plasma, (**f**) Ar purge, (**g**) precursor feeding, (**h**) Ar purge, (**i**) O_2_ plasma, and (**j**) Ar purge. To clearly describe the precursor chemisorption beneath the trench pattern and surface, the precursor chemisorbed on the sidewall of trench is omitted.

**Figure 9:**
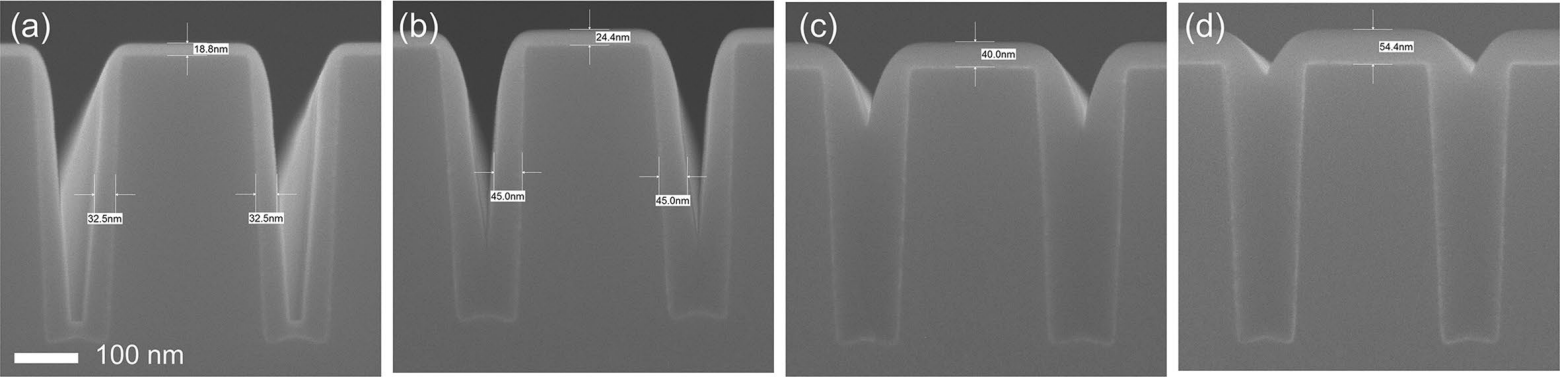
Cross-sectional FE-SEM images of the trench pattern (opening and depth of 150 and 400 nm, respectively) after conducting SiO_2_ PE-ALD using NH_3_^*^ for (**a**) 750, (**b**) 1000, (**c**) 1500, and (**d**) 2000 cycles.

**Figure 10:**
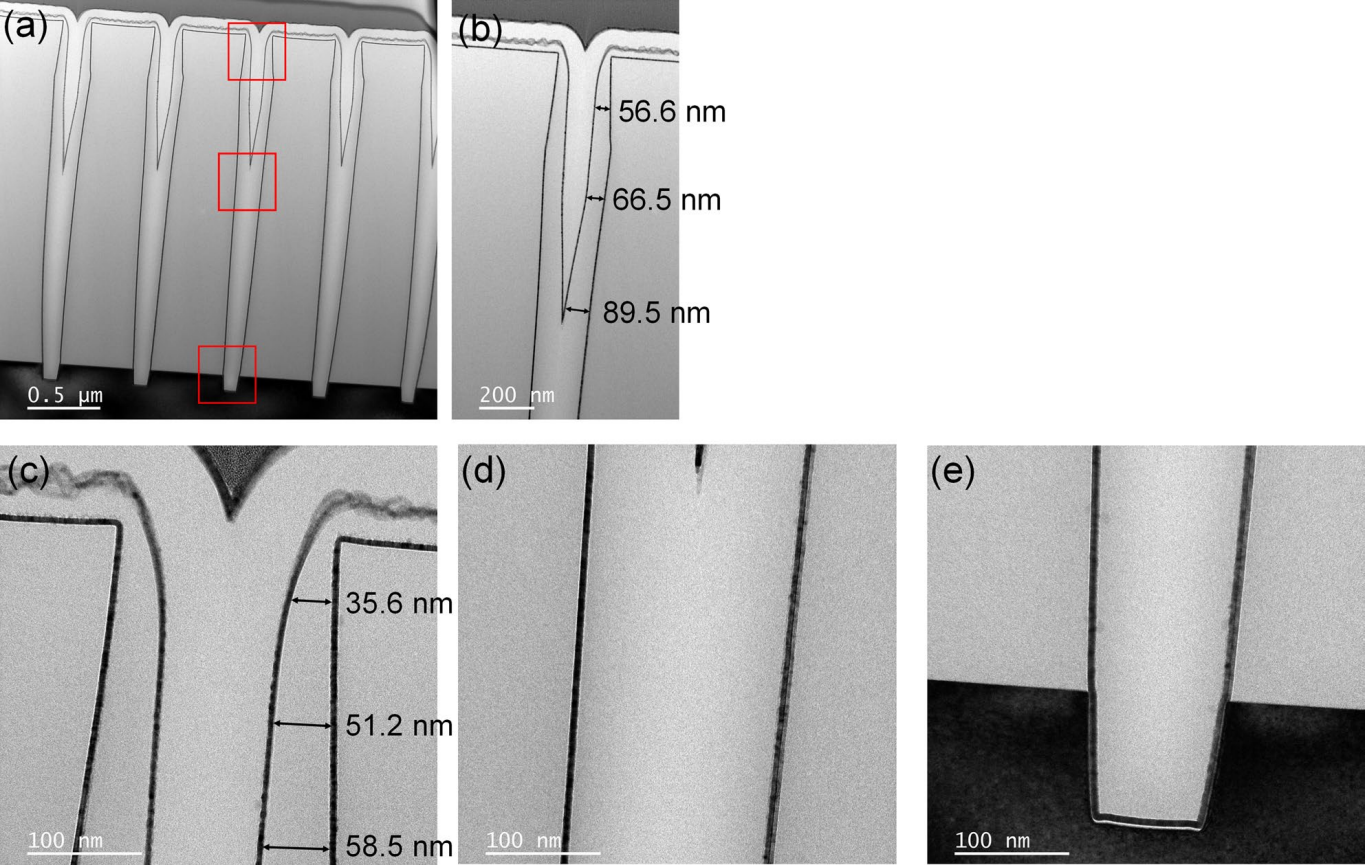
(**a**) Cross-sectional TEM image of the trench patterns (opening and depth of 200 nm and 2.5 μm, respectively) after conducting the SiO_2_ PE-ALD using NH_3_^*^. (**b**) 20,000× magnified image of trench opening area, and (**c**–**e**) 80,000× magnified images of (**c**) trench opening area, (**d**) center of trench, and (**e**) bottom of trench (depicted as red boxes in (**a**)), respectively.

These results confirmed that NH_3_^*^ can suppress the growth of SiO_2_ thin films deposited via PE-ALD by inactivating the Si–OH functional groups on the surface. NH_3_^*^ induces bottom-up growth behavior during the SiO_2_ PE-ALD process. In the case of no inhibitor (Fig. 8a–d), the introduced Si precursors were chemisorbed on the top and bottom of the trench-patterned structure (Fig. 8a) and exhibited the same growth rate. However, after NH_3_^*^ was performed (Fig. 8e–j), the inhibition only affected the top of the pattern (Fig. 8e), where the NH_3_^*^ was exposed; in turn, the growth was only inhibited on the surface and the top region (Fig. 8g). Therefore, the growth rate difference between the top and bottom regions of the pattern demonstrated bottom-up growth behavior (Fig. 8j). The inhibition of SiO_2_ on the top region of the pattern by the NH_3_^*^ treatment was confirmed by performing a cross-sectional SEM analysis of the trench pattern (Fig. 9). For NH_3_^*^, the thickness of the deposited SiO_2_ thin film on surface was 18.8 nm, whereas the thickness of SiO_2_ deposited on the side was 32.5 nm. As the deposition was conducted, the bottom-up growth of SiO_2_ in the trench pattern was observed. Furthermore, the bottom-up gap-fill characteristic was examined in a trench pattern with a small opening size and high aspect-ratio (opening and depth of 200 nm and 2.5 μm, respectively) after conducting SiO_2_ gap-fill using NH_3_^*^ by using the transmission electron microscopy (TEM) observation (Fig. 10). As shown in Fig. 10a, the trench was filled with SiO_2_ from the bottom of the pattern to the top region of the pattern. The thicknesses of SiO_2_ on the side where the region not filled (Fig. 10b) was gradually decreased from the bottom of 89.5 nm to the top of 56.6 nm also indicating the bottom-up characteristic. Moreover, at the negative sloped area near the top area of the trench (Fig. 10c), the deposited SiO_2_ film thickness gradually increased from the top (35.6 nm) to the bottom (58.5 nm). This is because of the relatively poor step coverage of NH_3_^*^ treatment. Due to the negative slope, the NH_3_^*^ was hard to reach on the surface of the side near the top area of the trench, inducing a gradient of the Si–OH functional groups on the surface. From the images of Fig. 10d,e, no seam was observed even in the TEM observation, indicating that the bottom-up gap-fill characteristics were achieved.

**Figure 11:**
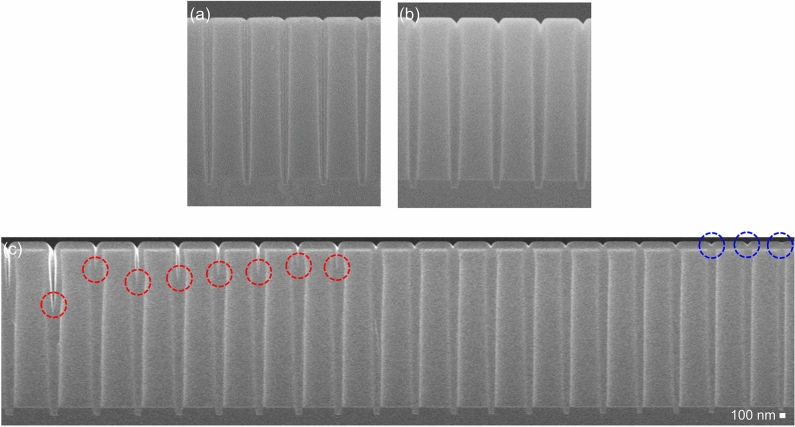
Cross-sectional FE-SEM images of the trench pattern (opening and depth of 200 nm and 2.5 μm, respectively) after conducting SiO_2_ PE-ALD with (**a**) no inhibitor and (**b**) NH_3_^*^, followed by wet etching (diluted HF of 200:1 for 60 s). (**c**) Cross-sectional FE-SEM images after conducting SiO_2_ PE-ALD with NH_3_^*^ on the trench pattern with a depth of 3 μm and an opening size varying from 300 (left) to 100 (right) nm.

To confirm the seamless gap-fill characteristics more accurately, wet etching (using an etchant of diluted HF at a ratio of 200:1 for 60 s) was conducted, followed by cross-sectional SEM analyses. As shown in Fig. 11a, the seam propagates to the bottom of the pattern because of the infiltrated etchant in the case of the SiO_2_ thin film deposited with no inhibitor. In contrast, in the case of NH_3_^*^, no seam is observed and only the surface is etched out with notch shapes (Fig. 11b). This profile indicates that no holes or voids are present where the etchant can infiltrate. In other words, perfect bottom-up growth was achieved by using NH_3_^*^. Owing to the bottom-up growth behavior, the gap-fill characteristics were significantly enhanced. The SiO_2_ PE-ALD process with NH_3_^*^ was performed on the patterned structure, which had a varied trench opening size (Fig. 11c). On the large-opening-size trench, the trench was not fully filled, but it showed the bottom-up growth behavior; the level of the end point of the unfilled region (indicated by red circles in Fig. 11c) gradually moved upward to the surface. This bottom-up growth resulted in perfect gap-fill characteristics in the narrowest trench. Moreover, bottom-up growth was observed even on the surface after completion of the gap fill of the trench (blue circles in Fig. 11c).

## Conclusion

The bottom-up growth of the SiO_2_ PE-ALD process was investigated by employing an inhibitor of NH_3_ plasma pre-treatment to demonstrate seam- and void-less gap fill on an extremely high-aspect-ratio pattern. Plasma pre-treatment with N_2_ or NH_3_ gas effectively decreased the growth of SiO_2_ PE-ALD without any contamination because of the suppression of the chemisorption of DIPAS on the substrate surface. The plasma concentration gradient in the trench structure induced a growth rate difference between beneath the trench and the surface, resulting in the relationship between enhanced gap-fill characteristics and the plasma pre-treatment process condition, which has a higher growth inhibitory effect. Owing to the inhibition effect of NH_3_^*^, the bottom-up growth behavior of SiO_2_ PE-ALD was successfully implemented in the trench structure. Finally, a seamless gap-fill process was achieved in the high-aspect-ratio pattern.

## Methods

SiO_2_ thin films were deposited by applying PE-ALD)(MAHA_AL, Wonik IPS) using DIPAS as the Si precursor and O_2_ plasma with capacitively coupled plasma at a frequency of 13.56 MHz as the oxygen source. The Si precursor was introduced using a bypass-type source delivery system with a heated canister at 50 °C and an Ar flow as the carrier gas. The PE-ALD process temperature was 50 °C, and the sequence consisted of precursor feeding, Ar purging, O_2_ plasma with a plasma power of 100 W, and Ar purging for 3, 5, 1, and 5 s.

Plasma pre-treatment was conducted prior to the SiO_2_ PE-ALD sequence to inhibit the growth of SiO_2_ PE-ALD. The plasma pre-treatment sequence consisted of gas feeding and plasma treatment, each for 1 s. Two types of gases, N_2_ and NH_3_, were employed, and the plasma pre-treatment was conducted at a process temperature of 50 °C with a plasma power of 100 W. The total deposition sequence is shown in Fig. 5.

The thicknesses of the deposited SiO_2_ thin films were measured by applying ellipsometry (Aleris, KLA-Tencor). The depth profiles, compositions, and chemical states of the thin films were analyzed using XPS (NEXSA, ThermoFisher Scientific). The gap-fill characteristics of the SiO_2_ PE-ALD process were examined by performing cross-sectional SEM (FE-SEM, JSM 760F, JEOL) and TEM (JEM-F200, JEOL) in trench structures having a depth of 1 μm and opening sizes varying from 100 to 300 nm.

## Data Availability

All data generated or analysed during this study are included in this published article.
